# Intraocular lens optic capture: a fixture for over 35 years

**DOI:** 10.3389/fopht.2026.1716870

**Published:** 2026-03-16

**Authors:** Brian M. DeBroff, Howard V. Gimbel

**Affiliations:** 1 Department of Ophthalmology and Visual Science, Yale University School of Medicine, New Haven, CT, United States; 2 Department of Ophthalmology, Loma Linda University, Loma Linda, CA, United States

**Keywords:** double optic capture, IOL capture, IOL fixation, optic capture, posterior capsulorhexis, posterior capsulorhexis with optic capture, reverse optic capture

## Abstract

The creation of a round, continuous curvilinear opening in the lens capsule has enabled techniques to fixate or capture the intraocular lens (IOL) optic through the opening to achieve fixation of the IOL. Optic capture enable IOL fixation and centration even in the absence of an intact capsular bag. This was first described for usage in the anterior capsule, but has subsequently been shown to be useful not only for the posterior capsule, but even for double fixation through intact curvilinear openings in both anterior and posterior capsules. This editorial reviews the many different techniques of optic capture that evolved over the last 35 years.

## 1 Introduction

With the evolution of the can-opener capsulotomy to the continuous curvilinear capsulorhexis (CCC) first described by Gimbel and Neuhann in 1990, the creation of tear resistant openings that resists radial tears while helping to insure placement of the intraocular lens within the capsular bag became the routine method in cataract surgery (1). Optic capture can only be performed when the capsulotomy opening is a smaller diameter than the IOL optic (ideally at least 1 mm). If a capsulotomy is very small, care needs to be taken to avoid zonular stress when capturing the optic. Careful placement first on one side and then final capture 180 degrees opposite, can lead to less stressful capture with reduced zonular stress. Optic capture, however has actually been recommend in cases of zonular laxity and has been demonstrated to reduce long term dislocation (2, 3). Femtosecond laser assisted capsulotomy can be very useful to ensure successful optic capture by creating a perfectly circled capsulotomy, perfectly centered, and of a known and specified diameter. Several companies are exploring the option to perform not only the anterior capsulotomy opening, but also the posterior capsule opening (4–8).

## 2 IOL capture through an intact anterior continous capsulotomy

### 2.1 Rhexis fixated IOL capture through an intact anterior capsular opening

**Figure 1:**
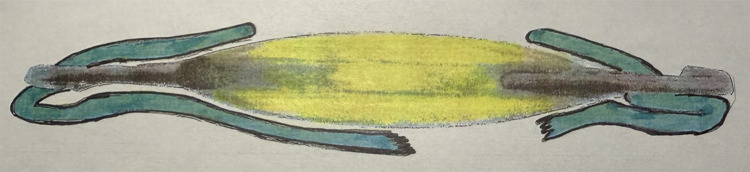
Rhexis fixated IOL: IOL haptics are in the sulcus and the IOL optic is captured posterior to the anterior capsulotomy opening.

The CCC also enabled the first description of capturing the IOL optic through the intact capsulorhexis in the event of a posterior capsule tear at the time of cataract surgery, a technique called the rhexis-fixated lens by Neuhann and Neuhann (9). This is a technique in which the haptics of a three-piece IOL are placed in the ciliary sulcus, and the IOL optic is prolapsed posteriorly through the CCC (**Figure 1**). This technique has also been referred to in the literature as IOL rhexis fixation (9, 10). Continuous curvilinear capsulotomy openings created using the Femtosecond laser also create a tear-resistant edge that is also amenable to rhexis fixation/IOL capture (8, 11). Gimbel and DeBroff later described using this technique in the case of a decentered IOL that needed to be repositioned, and also in the case when replacing an in-the-bag IOL after a Nd: YAG capsulotomy has been performed. The repositioned or new three-piece IOL can be placed with the haptics in the sulcus and the optic captured through the anterior capsulotomy opening or even through a scarred fibrotic capsule opening that, if necessary, has been enlarged using an anterior vitrectomy handpiece (10).

### 2.2 Reverse optic capture through an intact anterior capsular opening

**Figure 2:**
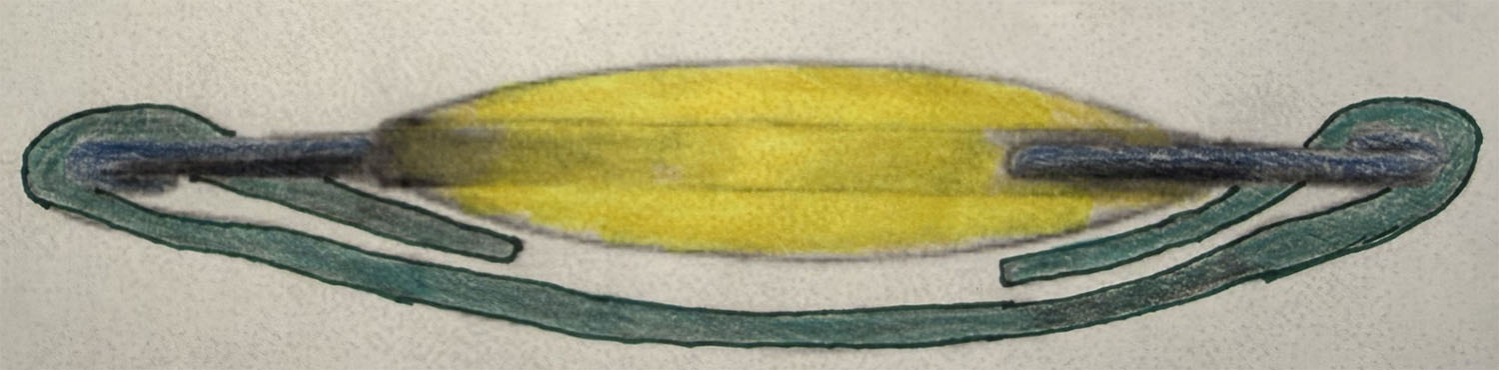
Reverse optic capture: IOL haptics are in the capsular bag and the IOL optic is captured anterior to the anterior capsulotomy opening.

An IOL placed within the capsular bag can have the optic placed anteriorly through an intact anterior CCC opening to achieve reverse optic capture (**Figure 2**). Both 3-piece IOL’s and one piece acrylic IOL’s can be reversed captured. The reverse optic technique is technically less difficult with a 3-piece IOL.

#### 2.2.1 To treat negative dysphotopsia

Samuel Masket found that negative dysphotopsia can be reduced, eliminated, or prevented by reverse optic capture (12). When the IOL optic is anterior to the anterior capsulotomy opening rather than behind it, the light rays are altered, helping to eliminate the light-bending distortions.

#### 2.2.2 Fixation of an IOL in the setting of non-intact posterior capsule

Gimbel and DeBroff reported that reverse optic capture can be utilized in cases when a posterior capsule tear either occurs after the IOL is placed in the bag (10). Also in cases in which a PC tear is not noticed or extends during IOL placement, reverse optic capture can be utilized to secure the one piece IOL. When a posterior capsule tear occurs before IOL implantation, reverse optic capture can still be utilized to implant a one-piece IOL and thus avoiding placement of the haptics in the sulcus. Jones et al. published a series of 16 patients in the setting of posterior capsule tear, a one piece IOL was injected through the incision, the haptics were placed posterior to the anterior CCC, and the optic was reverse optic captured through the anterior CCC (13). The technique of performing reverse optic capture has been described by Jason Jones by positioning a spatula or a Kuglen hook through the main incision reaching underneath the optic and then vaulting the entire optic forward through the opening of the intact anterior capsulotomy (14). Such a technique requires delicate manipulation and secure capturing of the IOL optic to prevent posterior dislocation of the IOL into the vitreous. In such a manner, the optic is entrapped centrally in front of the bag while the haptics remain in what is left of the capsular bag. OVD needs to be aspirated slowly to avoid loss of reverse optic capture.

In cases of posteriorly dislocated IOL’s, surgeons performing pars plana vitrectomy can utilize reverse optic capture once the IOL is brought anteriorly and it can subsequently be captured through an intact anterior capsulotomy to achieve fixation (15).

#### 2.2.3 Correction of residual hyperopia after in-the-bag IOL placement

The technique of reverse optic capture has been demonstrated in a series of 16 eyes by Akaishi et al. as a method to correct residual hyperopia after in-the-bag placement of a multifocal IOL (16).

#### 2.2.4 Prevention of rotation of a toric IOL

Gimbel published a case of reverse optic capture utilized to stabilize a toric IOL (17). After rotating the in-the-bag IOL to the proper axis, the IOL optic is captured through the anterior CCC to prevent post-operative IOL rotation. Reverse optic capture can also address the difficulty of toric IOL’s that tend to rotate either at the time of surgery or post-operatively due to a large capsular bag (17).

#### 2.2.5 IOL stabilization in cases of zonular weakness

Capsular tension rings may improve zonular stability, but are often insufficient in cases with more significant zonular compromise (18). Reverse optic capture provides additional stabilization by anchoring the optic against the anterior capsule rim (19).

## 3 IOL capture through an intact posterior capsulotomy

### 3.1 Haptics in sulcus and IOL captured through an intact posterior capsular opening

**Figure 3:**
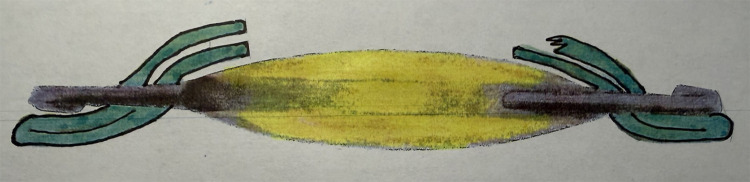
Posterior capsulotomy with optic capture: IOL haptics are in the ciliary sulcus due to break in anterior capsule rim and the IOL optic is captured through a posterior capsulotomy.

The technique of placing the IOL haptics in the ciliary sulcus and capturing the optic through an intact posterior capsulotomy (**Figure 3**) is useful in cases for which placement of haptics in the capsular bag is impossible (for example due to an extension of an anterior capsulorhexis, break in the anterior capsular rim during cataract removal, or discontinuous/can-opener/compromised anterior capsule rim), but there is the need to ensure IOL centration and prevent posterior capsular opacification. For this technique, an intact posterior continuous curvilinear capsulorhexis (PCCC) is required. This can be achieved via a primary PCCC or a small posterior capsule tear that is converted to a PCCC (20). Vasavada reports this technique to be useful in pediatric cases because it decreases the total area of optic contact with the iris, reducing the uveal inflammatory response (21).

### 3.2 Haptic in capsular bag and IOL captured through an intact posterior capsular opening

**Figure 4:**
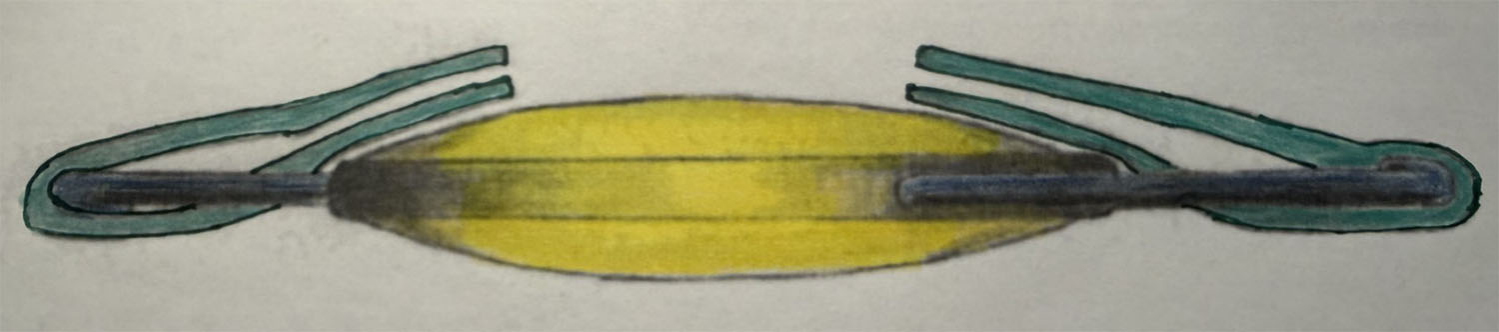
Posterior capsulorhexis with optic capture: IOL haptics are in the capsular bag and the IOL optic is captured through a posterior capsulotomy.

This technique of posterior capsulorhexis with optic capture (**Figure 4**) was originally described in 1994 by Gimbel and DeBroff to maintain a clear visual axis after pediatric cataract surgery (22). This technique involves capturing the IOL optic through a primary PCCC while the haptics remain within the capsular bag. Numerous studies have demonstrated this technique to be a valuable method to prevent secondary cataract formation and to maintain excellent IOL centration for children under the age of 6 in whom a primary posterior capsulotomy is advocated (23–30).

Performing an anterior vitrectomy in conjunction with this technique is optional. Some studies have demonstrated that an anterior vitrectomy done routinely in conjunction with PCCC with optic capture may be beneficial, especially in children under the age of 5 years (20, 31, 32), and as a method to improve low-contrast sensitivity (33). A prospective randomized study by Raina et al, however, demonstrated that PCCC with optic capture prevents secondary opacification of the visual axis in the absence of an anterior vitrectomy (34). In 2006, Rupert Menapace described a technique of hyaloid sparing posterior capsulorhexis with optic capture in adults (35). By utilizing a cohesive ocular viscoelastic device (OVD) to separate the capsule from the anterior hyaloid and thus preserving Berger’s space, no vitrectomy and no posterior capsule opacification occurred in 500 consecutive patients (35). Menapace called this variant of posterior capsulorhexis with optic capture: “posterior capsulorhexis with buttonholing”, and promoted it as a method to routinely treat adult cataracts and potentially eliminating posterior capsular opacity.

### 3.3 Reverse optic capture through an intact anterior, posterior, or fibrous capsular opening

**Figure 5:**
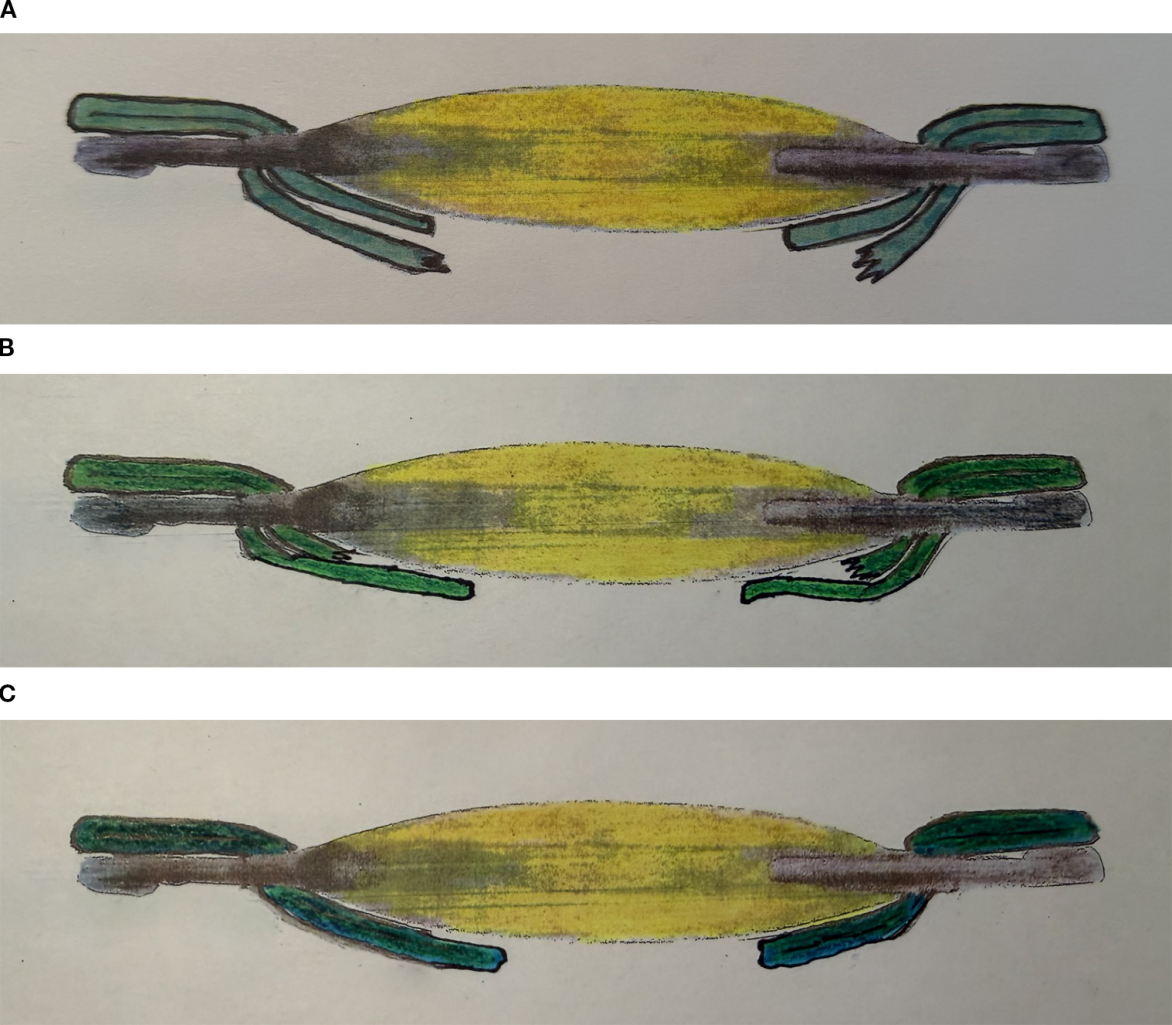
**(A)** Reverse Optic Capture: IOL haptics are posterior to the capsular support and IOL optic is reverse captured through an intact anterior capsulotomy. **(B)** Reverse Optic Capture: IOL haptics are posterior to the capsular support and IOL optic is reverse captured through an intact posterior capsulotomy. **(C)** Reverse Optic Capture: IOL haptics are posterior to the capsular support and IOL optic is reverse captured through a capsular membrane support.

Reverse optic capture can be utilized after posterior IOL decentration, tilt, subluxation, or dislocation. When a surgeon encounters posterior capsule tear, often a sulcus IOL may be placed at the time of surgery. If there is inadequate capsular support, zonular instability, or inadequate haptic length, the patient may present with a shift or complete dislocation of the IOL posteriorly (15). These cases often require either anterior vitrectomy or pars plana vitrectomy. Methods to successfully place an implant include IOL replacement, IOL suturing, scleral fixation of an IOL. In cases where there exists an intact capsulotomy opening (either anterior or posterior) or a fibrous capsular opening at least 1 mm smaller than the IOL optic, the technique of reverse optic capture may be utilized to stabilize the IOL. After either pars plana vitrectomy or anterior vitrectomy is used to free all vitreous strands and prolapse, the IOL can be brought forward and the IOL optic is captured through the capsular opening while the haptics remain posterior to the capsular membrane support (10, 36, 37). The IOL optic can be captured through an intact anterior capsulotomy (**Figure 5A**), an intact posterior capsulotomy (**Figure 5B**), or through a fibrous capsular opening (**Figure 5C**).

If the capsule or membrane opening is too small, a larger size opening can be created with the anterior vitrectomy handpiece. Care must be maintained when removing OVD to avoid loss of reverse optic capture.

## 4 IOL capture through both an intact anterior and posterior capsulotomy (double optic capture)

**Figure 6:**
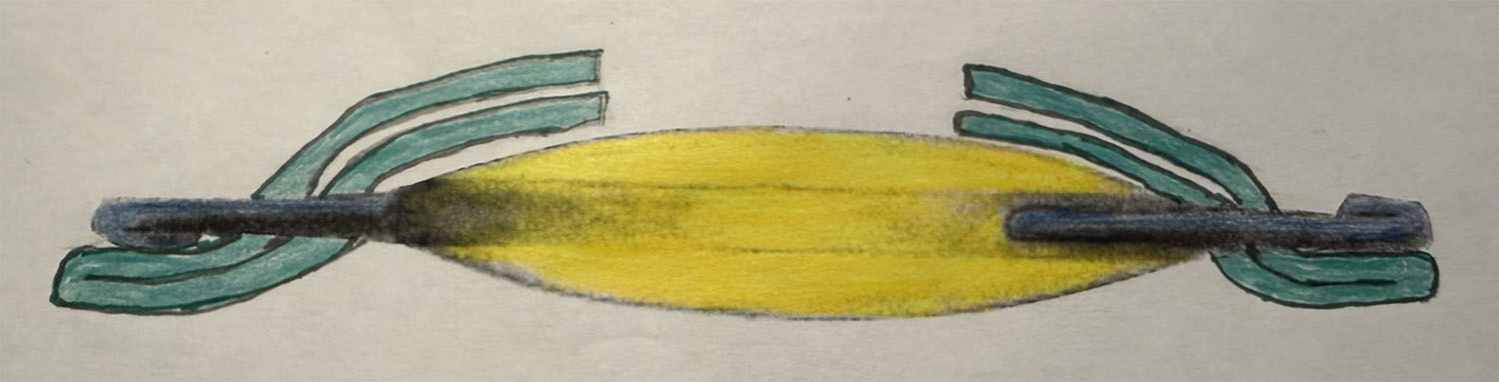
Double optic capture: IOL haptics are in the ciliary sulcus and the IOL optic is captured through both the anterior capsulotomy and the posterior capsulotomy.

Double optic capture was first described in 2008 by DeBroff and Nihalani as a method to reduce visual axis opacification while ensuring IOL centration in pediatric cataract surgery (38). Double optic capture is achieved by performing an anterior CCC, removal of the cataract, performing a posterior capsule opening (with either a vitrectomy handpiece or a posterior CCC), anterior vitrectomy if needed, placement of the three-piece IOL haptics in the ciliary sulcus, followed by capture of the IOL optic through both the anterior and posterior capsule openings (5, 38–40) (**Figure 6**). This technique leads to fusion of the anterior and posterior leaflets of the capsular bag 360 degrees, effectively sealing the capsular bag. Double optic capture has been demonstrated in long term follow-up studies to maintain IOL stability and clarity of the visual axis (41–43). 

With double optic capture the haptics are in the sulcus. It has been shown that due to the secure and stable fixation of the IOL, there is minimal haptic rub of uveal tissues and a low incidence of persistent anterior chamber inflammation postoperatively (4). In fact, Shah et al. presented a case of IOL exchange of a sulcus placed single piece IOL what was causing uveitis, glaucoma, hyphema (UGH) syndrome. After removal of the single-piece IOL, a three-piece IOL was placed in the sulcus and the optic was captured through both the anterior and posterior capsulotomy openings (44, 45). This demonstrated use of double optic capture to treat an inflammatory, acute uveitis condition confirms the ability of this technique to stabilize the IOL and prevent uveal chaffing even with placement of the haptics within the ciliary sulcus. More recent studies have also demonstrated optic capture is effective in cases of significant zonulopathy leading to a very stable IOL fixation (46). It should be noted that a three-piece IOL is recommended when performing double optic capture, as one piece acrylic IOL haptics should be avoided in the ciliary sulcus. If the IOL loses it captured position, there would be a higher incidence of posterior capsular fibrosis or anterior vitreous membranes which could necessitate either laser treatment or a secondary procedure to clear the visual axis.

Once the anterior and posterior capsules are opposed with capture techniques, IOL capture becomes even more secure and fibrosis of the capsule leaflets will prevent separation. If future IOL exchange is needed, DeBroff in a recent publication demonstrated successful removal of the IOL without sacrificing or compromising capsular support and successful re-capturing of the IOL optic through the fused capsular opening 17 years after the initial double optic capture procedure (47).

DeBroff described a case of surgical treatment of a unilateral congenital cataract with persistent hyperplastic primary vitreous (PHPV), now often referred to as Persistent fetal vasulature (PFV) by using the technique of double optic capture, DeBroff was able to achieve a clear visual axis and 20/20-2 vision after 20 years follow-up in a case of surgical treatment of a unilateral congenital cataract with persistent hyperplastic primary vitreous, also known as persistent fetal vasculature (47). Also, Dr. Howard Gimbel presented a case of double optic capture utilizing posterior capsulorhexis without anterior vitrectomy to successfully treat a congenital pediatric cataract associated with Down’s Syndrome (48). Arbisser in 2022 described using double optic capture with Menapace’s hyaloid sparing technique (49) as a method to avoid posterior capsular opacity in routine adult cataract cases (49, 50).

## 5 Summary

Since the creation of a continuous curvilinear opening in the capsule, either anterior, posterior, or both, ophthalmologists have created methods to secure the IOL to the capsule for stability, preventing uveal chaffing, and to discourage or prevent posterior capsular opacity. By securing the IOL, maintaining a two-chamber eye, and sealing residual lens epithelial cells, this technique has improved surgical outcomes over many years. This paper has demonstrated optic capture through an anterior capsulotomy, a posterior capsulotomy, and through both anterior and posterior capsulotomies. In addition the different optic capture techniques for each variety along with their indication have been presented. (**Table 1**) We have come a long way from the initial optic capture summary paper by Gimbel and DeBroff in 2004 (10), and future techniques will continue to be developed. New surgical techniques and specially designed IOL’s will inevitably lead to expanded applications and techniques of optic capture in the future.

**Table 1:** Types of IOL capture, the different techniques to perform optic capture, and their indications.

Type of IOL capture	Technique	Indication
IOL capture through an anterior capsulotomy	Rhexis fixation	Management of a posterior capsular tear
	Reverse optic capture	Treatment of negative dysphotopsia
		Fixation when there is rupture of the posterior capsule
		Correction of residual hyperopia
		Prevention of toric IOL rotation
		Stabilization of IOL in cases of zonular weakness
IOL capture through a posterior capsulotomy	Haptics in the sulcus	Cases of compromised anterior capsular rim
	Haptics in the capsular bag	Prevention of secondary membranes in pediatric cataract surgery (posterior capsulorhexis with optic capture)
	Reverse optic capture	Management of unstabile, tilted, posterior subluxated, or dislocated IOL
IOL capture through both anterior and posterior capsulotomy	Double optic capture	Prevention of posterior capsular opacity in children and adults
